# A Qualitative Study of Pharmacists’ Perceptions of the Advantages and Disadvantages of Telepharmacy

**DOI:** 10.3390/pharmacy12060169

**Published:** 2024-11-16

**Authors:** Masaki Shoji, Mitsuko Onda

**Affiliations:** Department of Social and Administrative Pharmacy, Faculty of Pharmacy, Osaka Medical and Pharmaceutical University, Osaka 569-1094, Japan

**Keywords:** telepharmacy, telemedicine, community pharmacy, medical communication

## Abstract

In Japan, telepharmacy is becoming increasingly popular due to deregulation triggered by the outbreak of COVID-19. The purpose of this study was to gain an understanding of the actual state of telepharmacy in Japan by interviewing pharmacists who have experience with telepharmacy and describing its advantages and disadvantages, as well as their outlook for its use going forward. The interviews were conducted online using Zoom. Each interview lasted approximately 30 min. Eleven people were interviewed. The advantages mentioned by the pharmacists were classified into three main categories: “Better communication”, “Time savings”, and “Improved safety”. The disadvantages were classified into the following nine categories: “Drug delivery problems”, “Communication failures”, “Ease of use for patients”, “Emotional reactions”, “Pharmacy system”, “Communication issues”, “Healthcare system issues”, “App system issues”, and “Cost”. Many of these factors correspond to the Unified Theory of Acceptance and Use of Technology (UTAUT) constructs presented by Venkatesh, et al. Many of the pharmacists mentioned that the use of telepharmacy is likely to expand further in the future, but that this will require further development of communication technology and the widespread use of systems such as electronic prescriptions.

## 1. Introduction

Telemedicine has been developed primarily as a way to alleviate the shortage of medical resources in rural areas, and its advantages and disadvantages have been studied in many countries [1,2,3]. In Japan, telepharmacy has been practiced since 2000 in accordance with the “Summary of Reforms to Systems Including the Law for Ensuring the Quality, Efficacy, and Safety of Drugs and Medical Devices” notification that was issued in 2018. This notification placed many restrictions on the practice of telepharmacy, including a requirement that the initial counseling session be provided in person [4]. With the outbreak of the COVID-19 pandemic, however, telepharmacy attracted attention as one a way to avoid the spread of infection in medical facilities [5,6,7]. In Japan, the Ministry of Health, Labour, and Welfare (MHLW) issued the notification “On the Temporary and Exceptional Handling of Medical Treatment Using Telephones and Information and Communication Equipment in Connection With the Spread of the COVID-19 Pandemic”. This was called the 0410 response, and implemented deregulatory actions such as allowing telepharmacy even for first-time patients and making all drugs, in principle, eligible for telepharmacy. Although it has been reported that pharmacists feel that the 0410 response has made it easier to provide telepharmacy [8], this special measure was actually utilized in only 0.51% of the 68,849 total cases during May and June of 2020 [9]. In 2022, the Order for Enforcement of the Act on Securing the Quality, Efficacy, and Safety of Products Including Pharmaceuticals and Medical Devices was revised, and many of the requirements regarding the practice of telepharmacy from the 0410 response were kept, but some changes were also made, such as a requirement that video conferencing be used [10].

As described above, the regulations regarding telepharmacy have continued to evolve. While there have been scattered surveys on telepharmacy in Japan, including a survey of patient awareness [11] and a study of the requirements for promoting awareness based thereon [12,13], to the best of the authors’ knowledge, no detailed investigations of pharmacists’ perceptions and perspectives have been conducted. The purpose of this study was to interview pharmacists who have actual experience with telepharmacy about their perceptions and perspectives and interpret them based on existing models. We believe that by aggregating pharmacists’ actual experiences with and perspectives on telepharmacy, we will be able to gain deeper insights into the safety of pharmacotherapy and healthcare systems in general.

## 2. Materials and Methods

### 2.1. Study Design

This study was an exploratory study that was based on an interpretivist perspective. Semi-structured interviews were conducted with pharmacists who had experience providing telepharmacy. In addition, this study employed thematic analysis and the hybrid approach combining deductive and inductive analytical methods that was presented by Braun and Clark [14]. Specifically, we first conducted a functional analysis on the data obtained through interviews, and then attempted to apply the data to existing theories.

### 2.2. Interview Method and Contents

All of the interviews were conducted online using Zoom (Zoom Video Communications), once per interviewee, between October and November 2023, by the corresponding author. We asked three pharmacy chain companies that proactively practice telepharmacy to select interviewees, and also reached out through social networking services to pharmacists with experience in telepharmacy. Each interview lasted about 30 min, and was conducted using either the interviewee’s workplace or home computer. The interviews were recorded with the interviewees’ consent, transcribed, and subjected to thematic analysis. The questions were as follows: 1. pharmacists’ work experience; 2. main categories of users of telepharmacy; 3. advantages of telepharmacy; 4. disadvantages of telepharmacy.

### 2.3. Analysis Methods

The interviewees were anonymized during the transcription process so that their identities could not be identified or inferred.

**Figure 1:**
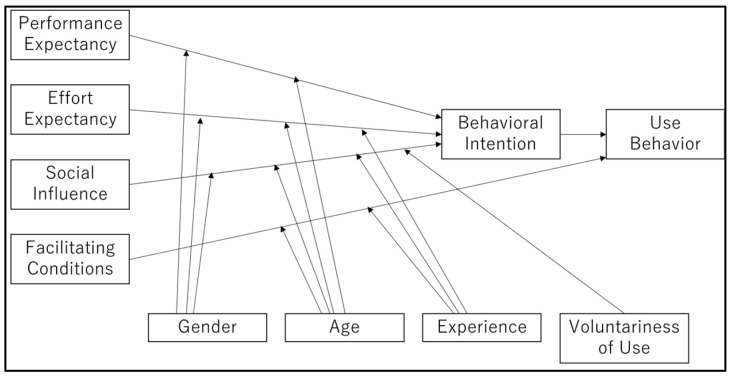
Unified Theory of Acceptance and Use of Technology (UTAUT) [15].

The data analysis in the inductive analysis step of this study was performed according to the six-step guide to thematic analysis presented by Braun and Clark. Specifically, in the first step, all researchers reviewed the transcribed data and made sure they fully understood the contents. In the second step, coding was performed, in which the textual data were segmented into smaller units along the lines of actions, events, thoughts, etc., and coded. In the third step, themes representing more abstract concepts were identified from the results of the coding of the segmented text data. The fourth step was a review of the theme names to ensure that the themes established in the third step were consistent with the coding process, which included interpretations of the textual data. In the fifth step, the lead author established the final themes and reinterpreted them using as a framework the Unified Theory of Acceptance and Use of Technology (UTAUT) (Figure 1) presented by Venkatesh, et al. [15]. In the sixth step, lead author made a report of the findings and share with the co-author. All coding work was conducted using NVivo 1.7.2 (QSR International) qualitative data analysis software. The Japanese version of the Standards for Reporting Qualitative Research (SRQR) [16] was used when writing the paper.

### 2.4. The Unified Theory of Acceptance and Use of Technology (UTAUT)

The UTAUT organizes the factors of technology acceptance into “Performance Expectancy”, “Effort Expectancy”, “Social Influence”, and “Facilitating Conditions”. The moderators are “Gender”, “Age”, “Experience”, and “Voluntariness of Use” [15]. Currently, the UTAUT is the dominant technology acceptance model, and is used in a variety of fields [17].

## 3. Results

The backgrounds of the interviewees are shown in Table 1. Of the 11 interviewees, 6 were male and 5 were female. The average number of years of employment as a pharmacist was 10.3 (maximum 21, minimum 1). Seven pharmacists practice telepharmacy regularly, and the other four irregularly.

Table 2 shows the patient demographics that interviewees cited as common among users of telepharmacy. Most of the diseases were chronic diseases such as lifestyle-related diseases, and most of the patients were in their 40–50s.

### 3.1. Advantages of Telepharmacy

The advantages of telepharmacy were classified into three categories: “Better communication”, “Time savings”, and “Improved safety” (Table 3).

#### 3.1.1. Better Communication

This category contained five sub-categories: “Able to talk in a relaxed fashion”, “Able to talk to a person who is assisting the patient with his/her daily life”, “Able to concentrate on the medication counseling”, “A high degree of privacy”, “Easier to refer to information on the internet during medication counseling”, and “Easier to check on the patient’s medication adherence”. In the interviews, pharmacists talked about how telepharmacy can improve the quality of communication by providing a more relaxed environment, including the participation of the patient’s family, and the ability to more easily check if there are any leftover medications.

#### 3.1.2. Time Savings

This advantage was most commonly mentioned by interviewees as an advantage of telepharmacy. In the interviews, the pharmacists mentioned that telepharmacy allows patients to pick up their medications without physically coming to the pharmacy, thus avoiding travel and waiting time.

#### 3.1.3. Improved Safety

This category may be broken down into two further sub-categories: “Protection from infection” and “Avoidance of travel-related risks”. Pharmacists mentioned as an advantage of telepharmacy the ability to ensure the safety of both the patients and the pharmacists from infectious diseases such as COVID-19 and from the danger of suffering heat stroke in the summer.

### 3.2. Disadvantages of Telepharmacy

The disadvantages of telepharmacy were grouped into nine categories: “Drug delivery problems”, “Communication failures”, “Ease of use for patients”, “Emotional reactions”, “Pharmacy system”, “Communication issues”, “Healthcare system issues”, “App system issues”, and “Cost” (Table 4).

#### 3.2.1. Drug Delivery Problems

Many pharmacists mentioned “delays in patients receiving their medications”. In the telepharmacy system, pharmacists mail medications to patients’ homes after completing an online medication instruction session. Patients must wait several days for the medication to be delivered to their homes after the instruction. Also, some pharmacists mentioned concerns about accuracy and the possibility of medicines being damaged during shipping. From this perspective, telepharmacy is not suitable for diseases that resolve in a few days.

#### 3.2.2. Communication Failures

The most frequently mentioned drawbacks of telepharmacy were “Communication failures”. Of the factors contributing to communication failures, the one that was mentioned most often was the stability of patients’ telecommunications equipment.

#### 3.2.3. Ease of Use for Patient

This category contained five sub-categories: “Difficult for the elderly”, “Difficulty in using a smartphone”, “Difficulty in using the app”, “Difficulty in video calling”, and “Difficult for patients with disabilities”. The practice of telepharmacy requires patient literacy in the use of these devices and systems. If a patient is unable to handle these tasks alone, an environment is needed in which the patient can obtain help from a family member or other helper. In addition, one pharmacist mentioned the importance of developing devices that are easy to use for patients with disabilities such as paralysis.

#### 3.2.4. Emotional Reactions

This category contained the following four sub-categories: “Concerns about registering a credit card”, “Unwillingness to try new things”, and “Resistance to show their house room”. These sub-categories could be considered related to risk awareness, motivation, and feelings of shame, rather than literacy.

#### 3.2.5. Pharmacy System

Some pharmacists mentioned the difficulty of performing their work in the pharmacy at the same time. During times of heavy patient traffic, they tended to prioritize responding to the patients who were there in person over their telepharmacy work, which sometimes resulted in them not being able to start their sessions of telepharmacy at the scheduled times.

#### 3.2.6. Communication Issues

This category consisted of the following four sub-categories: “Difficulty understanding patients’ facial expressions”, “Nervousness”, “Difficulty using the device to explaining medications”, and “Difficulty to get a feel for patient’s moods”.

In the interviews, the small size of the screen was mentioned as a factor contributing to the difficulty in understanding patients’ facial expressions. In the sub-category “Nervousness”, patients’ unfamiliarity with telepharmacy was mentioned, somewhat contradicting the interviewee’s comments in “Better Communication” regarding the advantages of telepharmacy.

#### 3.2.7. Healthcare System Issues

The need to bring prescriptions to a pharmacy was mentioned as a healthcare system problem. Some pharmacists also suggested that the widespread use of electronic prescriptions to solve this problem would lead to telepharmacy becoming more widely adopted.

#### 3.2.8. App System Issues

Some pharmacists mentioned about “Difficulty of asking prescription inquiries” and “Difficulty of obtaining information about concomitant medications”. We hope that these issues will be resolved through application updates.

#### 3.2.9. Cost

Several pharmacists brought up the fact that patients have to pay the shipping costs. They said that some patients come to the pharmacy to pick up their medications to save on shipping costs. A small number of respondents also brought up the cost of setting up and running the telepharmacy system, and said they wanted it to be eligible for additional insurance points. In order to promote the more widespread use of telepharmacy, it will likely be necessary to create a framework that reduces the initial investment required and allows the developers to benefit.

## 4. Discussion

In this study, we interviewed pharmacists with experience with telepharmacy and analyzed their perspectives on the advantages and disadvantages of telepharmacy. The key findings of this survey were to identify issues that would not be immediately apparent without actual pharmacist experience, such as “Pharmacy system”, “Healthcare system issues”, and “App system issues”.

### 4.1. Advantages of Telepharmacy

The most frequently mentioned advantage of telepharmacy was “Time savings”. Patients who are too busy with work to take the time to visit the pharmacy will probably welcome telepharmacy, which can be conducted from anywhere. Surveys of patients have shown that patients also think “Time savings” is an advantage of telepharmacy [11,12,13], which shows that patients and pharmacists are in agreement on this issue.

There were conflicting comments about “Better communication” and “Communication issues”. For example, while several pharmacists stated that “It is possible to talk in a relaxed fashion”, others mentioned “Nervousness”, suggesting that there is a need to become familiar with telepharmacy itself.

It is noteworthy that many pharmacists mentioned being “Able to talk to a person who is assisting the patient with his/her daily life”. This would improve the quality of daily care and lead to improved patient medication adherence and the discovery of errors in usage of which the patients themselves had not been aware, or of the existence of any medicine that might be left over. Therefore, telepharmacy should be considered particularly when the patient is suspected of having cognitive impairment.

Thus, telepharmacy is not only a timesaver for busy younger adults, it also has the potential to improve the safety and effectiveness of patients’ medication by improving the quality of information exchange.

### 4.2. Disadvantages of Telepharmacy

The most frequently mentioned disadvantage of telepharmacy was “Communication failures”, with many pharmacists having experience with the video or audio cutting out. This was attributed to the fact that patients were using their own smartphones during telepharmacy sessions and were in locations with poor reception, such as rural areas, underground, or buildings that block signal. When pharmacists are educating patients about the use of telepharmacy, it is necessary for them to encourage the patients to be somewhere that has good reception.

Regarding the “Ease of use for patients” category, the respondents mentioned not only the methods of use of the devices and applications, but also the problems caused by the small screen size. A system that provides an expanded field of vision or makes it possible to easily zoom in and out while maintaining the same resolution is needed.

The authors think that the resistance of the elderly to registering their credit cards in apps may be due in part to potential wariness of fraud and leakage of personal information. One of the pharmacists interviewed mentioned that the absence of internet shopping experience may also have an impact. In fact, Ozeki et al. reported that more than 60% of older adults who had received telepharmacy preferred to come to the pharmacy to pick up their medications [13].

Regarding the “Pharmacy system” category, many pharmacists mentioned the difficulty of combining telepharmacy work with their regular work. Pharmacies typically assign pharmacists based on the number of patients who bring prescriptions into the pharmacy, and in this study, pharmacists tended to prioritize responding to outpatients. In addition, all pharmacists provided telepharmacy within the pharmacy where they worked. Since these factors make it difficult to start the telepharmacy sessions exactly at the times reserved by patients, it is necessary to consider making it easier for pharmacists to choose a more flexible and convenient location, such as making it easier for them to provide telepharmacy from their own homes. Looking at the “Healthcare system issues” category, several pharmacists mentioned “Expectations for electronic prescriptions”. Electronic prescriptions are a system to electronically dispense prescriptions, which are currently given on paper. In Japan, this system has been in operation since January 2023, but as of September 2024, while 48.5% of pharmacies had adopted the system, only 2.1% of hospitals had adopted it, indicating that the system has not fully penetrated the medical field [18]. The widespread use of electronic prescriptions will save patients the trouble of submitting their prescriptions to pharmacies, and will lead to the true realization of the most frequently cited advantage of “Time savings”.

Looking at “App system issues”, pharmacists mentioned the difficulty of inquiring about questionable prescriptions. In order to further promote the use of telepharmacy, we think it will be necessary to facilitate communication between the physicians who provide online medical care and the pharmacists who provide telepharmacy services.

Many pharmacists mentioned “Delays in patients receiving their medications” as an issue related to “Drug delivery problems”. As mentioned by the pharmacists in the interview, telepharmacy is not suitable for urgent cases, as it takes several days for patients to receive their medications. Sano et al. also reported that patients are of the opinion that they “can’t get the medication right away” [12]. Also, even in the case of chronic diseases, a requirement of telepharmacy is that the patient still has a certain quantity of medication remaining.

### 4.3. How Are These Issues Relevant to the UTAUT?

In this section, we discuss how the issues identified in this study can be correlated to the UTAUT.

Issues corresponding to “Performance Expectancy” would be issues related to the benefits and opportunities gained or lost from the use of telepharmacy. Therefore, the three advantages (“Better communication”, “Time savings”, and “Improved safety”) and the four disadvantages (“Drug delivery problems”, “Communication failures”, “Communication issues”, and “App system issues”) are considered to fall under the category of “Performance Expectancy”.

Issues corresponding to “Effort Expectancy” are the difficulties that arise when practicing telepharmacy, including “Ease of use for patients”, which suggests that the modifier “Age” may also be applicable.

The “Social Influence” category is the concept of how the use of the technology is perceived by people who are important to the users, such as the users’ colleagues. The category that comes closest to this for which information was obtained in this study is considered the “Pharmacy system” category, when respondents talked about the difficulties they had balancing their telepharmacy work and their work with outpatients, which is carried out in collaboration with other pharmacists.

The “Facilitating conditions” category relates to individuals’ perceptions of the degree to which telepharmacy is supported by the organizational and technological infrastructure, which we believe correspond to the categories “Pharmacy system”, “Helathcare system issues”, “App system issues”, and “Cost”.

As described above, the responses obtained from the interviews conducted in this study can all be categorized into the constructs of the UTAUT.

We recognize the following limitations of this study. First, this study surveyed pharmacists’ opinions about the current state of telepharmacy under the current Japanese healthcare system and technology, and therefore cannot be said to be universally applicable. For example, the degree of logistics development, which varies from country to country, as well as citizens’ smartphone ownership, cannot be considered in this study. Second, the interviewee number was small. Third, although the UTAUT focuses on “Behavioral Intention”, it is the patients themselves who make the decision to use telepharmacy. Fourth, since all of the pharmacists interviewed in this study worked in city pharmacies, the results do not reflect the reality of the remote areas. Therefore, the educational activities and willingness of pharmacists could be dependent variables here.

Despite its limitations, we believe this study describes many realistic points that could only be made by a pharmacist who has actually experienced telepharmacy. In these respects, this study has a certain novelty as a description of the advantages and disadvantages of telepharmacy.

In the future, we would like to conduct research on pharmacists’ experiences of the advantages and disadvantages of telepharmacy in remote areas and its application to pharmacy education.

## 5. Conclusions

Although online medication guidance has some advantages, such as time savings, convenience, and enhanced communication, there are many systemic issues that need to be addressed under the current situation in Japan. In particular, pharmacists believe that the widespread use of electronic prescriptions is necessary.

## Figures and Tables

**Table 1:** Respondent Attributes.

Respondent	Years of Employment	Gender	Experience with Telepharmacy *
A	10	Female	Regular
B	1	Female	Regular
C	10	Male	Irregular
D	9	Male	Regular
E	21	Female	Irregular
F	5	Female	Regular
G	12	Male	Regular
H	10	Male	Irregular
I	20	Female	Regular
J	9	Male	Irregular
K	6	Male	Regular

* Regular: Regularly provides counseling via telepharmacy, such as 1–2 times a week. Irregular: Honors occasional requests for telepharmacy services.

**Table 2:** Patients eligible for telepharmacy.

	Responses	N
Common diseases in users (multiple responses)	Chronic diseases (lifestyle diseases, etc.)	8
	COVID-19	2
	Psychiatric disorders	1
	Chemotherapy	1
Age group with the highest number of users	20–30s	2
	40–50s	6
	60–70s	1
	≥80s	2

**Table 3:** Advantages of telepharmacy.

Category (UTAUT Elements Relevant to the Category)	Sub-Category	Typical Comments (Responded Pharmacist)
Better communication (Performance expectancy)	Able to talk in a relaxed fashion	*The first thing I thought was that, you know, it is because the patients are at home. They are more relaxed than usual, and since it is one-on-one, it is easier to have an open, more in-depth conversation.* (PI)
	Able to talk to a person who is assisting the patient with his/her daily life	*In the case of elderly patients, it is ideal to have them listen to the medication instructions with their family members for many reasons. With medication counseling* via *telepharmacy, the patient can listen medication counseling with their family when they are available.* (PD)
	Able to concentrate on the medication counseling	*I feel that patinets are very conscious of what we are saying to them.* (PG)
	A high degree of privacy	*I feel that telepharmacy is more private.* (PF)
	Easier to refer information on the internet during medication counseling	*When a patient asks a question during medication instruction, I can refer the Internet for data, and also send them a pdf file I found.* (PG)
	Easier to check on patients’ medication adherence	*We can easily check how much medication they have left* (PI)
Time savings (Performance expectancy)	*I think a big advantage in that it places relatively few time constraints on both parties, and it eliminates waiting time. Patients don’t have to wait to pay, it can be done right away on credit, so there’s no loss of time for either party* (PK)	
Improved safety (Performance expectancy)	Protection from infection	*In cases of infection, such as COVID-19, if it’s online, patients and pharmacists both can feel safe.* (PH)
	Avoidance of travel-related risks	*It’s very hot right now, isn’t it? Everyone don’t want to go outside as much as possible. More and more elderly people are requesting delivery medicines because of the danger of heat stroke,* etc. *I think telepharmacy would be very convenient for such people.* (PI)

**Table 4:** Pharmacists’ perceived disadvantages of telepharmacy.

Theme (UTAUT Elements Relevant to the Theme)	Sub Theme	Typical Comments (Responded Pharmacist)
Drug delivery problems (performance expectancy)	Delays in patients receiving their medications	*We will deliver the medication, there is still a fairly long delay, you know. It may take a few days from the time of the counseling for the medicine to be delivered. I think that is the biggest drawback* (PH)
	Damage to the medicine while the shipping	*No matter how well it is packed, there is always a possibility that it could be damaged while shipping.* (PB)
	Concerns about accurate delivery	*Since I don’t hand it to the patients directly, I’m always a little anxious about whether or not they’ll actually get it.* (PB)
Communication failures (performance expectancy)	Communication failures	*Even though our connection is fairly stable, many of our patients are contacting us from their home using cell phones, not PCs. And if their cell phone signal is bad, they may cut in and out, or their picture might freeze.* (PJ)
	The session could be interrupted if a device runs out of battery	*There were people whose phones ran out of charge in the middle of medication instruction.* (PI)
Ease of use for patients (effort expectancy, experience)	Difficult for the elderly	*Since there are many elderly people, I feel that it is difficult to bring them to use the equipment until they are able to use it.* (PI)
	Difficulty in using a smartphone	*Many of the patients are elderly, so it is not only a question of whether or not they have a smartphone, but also how they actually operate it. I think that is the difficult part.* (PJ)
	Difficulty in using the app	*Many first-timers seem to get confused about how to make reservations, so I still call them individually and explain that I will send them a message that they can then use to make a reservation* (PG)
	Difficulty in video calling	*with people who are not so familiar with online communication, it is often difficult to understand what they are saying because their voice is very broken even though they are trying their best to speak.* (PG)
	Difficulty for patients with disabilities	*We also need to consider the case of people who have difficulty expressing themselves or moving their hands well because of their disease.* (PE)
Emotional reactions (behavioral intention, voluntariness of use)	Concerns about registering a credit card	*I think that the biggest bottleneck is the credit card registration.* (PC)
	Unwillingness to try new things	*The average age of people who receive medicine is also high, and I think that only a certain percentage of people are willing to try new things because they have been doing it a certain way until now.* (PK)
	Resistance to show their house room	*I think some patients concern or feel shame about showing their house room* via *screen. I mean, I would probably feel that way.* (PA)
Pharmacy system (facilitating conditions, social Influence)	Difficulty coordinating telepharmacy with regular work	*There have been two or three times when I was too busy with my pharmacy work, even though it was time for an online counseling session. Those times, I apologized to the patient.*(PF)
	Staffing shortage	*Staffing shortage… that may be part of the problem.* (PH)
Communication issues (performance expectancy)	Difficulty understanding patients’ facial expressions	*I think the small screen makes it difficult to see patients’ facial expressions. It is also difficult to hear them.* (PK)
	Nervousness	*I have a strong feeling that elderly people may be a bit nervous and tense when talking online, so they may not be able to relax.* (PK)
	Difficulty using the device to explaining medications	*I think it is difficult for patients to get an idea of how to use devices, such as inhalers, because they cannot see the actual product.* (PD)
	Difficulty to get a feel for patients’ moods	*The sense and the vibe that you can only get in a face-to-face setting is also different from what you can get online, so the whole experience is just a little different.* (PJ)
Healthcare system issues (facilitating conditions)	Expectations for electronic prescriptions	*I think it will become very popular, given that electronic prescriptions are now starting to be used, and as they become more widespread, prescriptions can be given directly to pharmacies from hospitals, so pharmacies can prepare and deliver medications to patients based on those prescriptions, and patients will no longer have to come in to pharmacies in person* (PF)
	Health insurance points	*Think about the economics, I think that if insurance points aren’t awarded, we will be restricted timewise by the need to meet the patients’ schedules, so if absolutely no insurance points are awarded, and everything is up to the patients, it will be pretty tough. Practically speaking.* (PK)
App system issues (facilitating conditions, performance expectancy)	Difficulty of asking prescription inquiries	*One issue is that patients make appointments to fit their own schedules. There have been times when the hospital isn’t open then, or I couldn’t reach the doctor, and I couldn’t give the patients their medicine. If the patient is a normal outpatient, and lives nearby, then maybe there would be something I could do, but… This has caused me the biggest headaches.* (PH)
	Difficulty of obtaining information about concomitant medications	*Telepharmacy requires patients to input concomitant medications into the app themselves, or to tell the pharmacist that medications they are taking themselves. I think there are probably many patients who do not input concomitant medications into the app.* (PG)
Cost (facilitating conditions)	Shipping cost	*When we talk about shipping medication, most patient say like ‘No, no, I will come to pharmacy and pick the med up. It will be waste for paying shipping bill’.* (PE)
	Equipment fee	*We have to invest in equipment.* (PH)

## Data Availability

The datasets generated and/or analyzed during the current study are available from the corresponding author(s) on reasonable request.
